# Influence of the Graphene Filler Nature on the Morphology and Properties of Melt Blended EVOH Based Nanocomposites

**DOI:** 10.3390/polym13203546

**Published:** 2021-10-14

**Authors:** Anthony Blanchard, Fabrice Gouanvé, Eliane Espuche

**Affiliations:** Ingénierie des Matériaux Polymères, University Lyon, Université Lyon 1, CNRS UMR 5223, F-69622 Villeurbanne, France; anthony.blanchard@live.fr (A.B.); fabrice.gouanve@univ-lyon1.fr (F.G.)

**Keywords:** nanocomposites, graphene, microstructure, oxygen barrier, water vapor barrier, mechanical properties

## Abstract

In this study, ethylene vinyl alcohol (EVOH) nanocomposites elaborated by melt blending with four different fillers were investigated. Two graphene and two graphite fillers displaying various shapes were selected. The morphology, microstructure, thermal, mechanical, and barrier properties of the nanocomposite films prepared for 2 wt% fillers were analyzed with the aim to establish structure–function properties relationships. The nanocomposites properties significantly depended on the nature of the incorporated filler. The nanocomposite film prepared with the expanded graphite filler exhibited the highest Young modulus value (E = 1430 MPa) and the best barrier properties. Indeed, barrier properties, rarely studied at high water activities, evidenced a significant improvement with a decrease of the water vapor permeability by a factor 1.8 and of the oxygen permeabilities by a factor close to 2, for a critical water activity higher than 0.95. An increase of the thermal stability was also evidenced for this nanocomposite. It was shown that for all studied nanocomposites, the properties could be related to the dispersion state of the fillers and the simultaneous increase of the crystallinity of the matrix. A specific equation was proposed to take into account these both parameters to accurately predict the nanocomposite barrier properties.

## 1. Introduction

Widely used in food packaging, building, and automotive applications, ethylene vinyl alcohol (EVOH) copolymers are a family of semi-crystalline copolymers of ethylene and vinyl alcohol. These copolymers exhibit excellent barrier properties to oxygen, organic solvents, and food aromas in dry condition [1,2,3,4]. However, at high water activities, their significant moisture sensitivity leads to the deterioration of their gas barrier properties. As a consequence, many researches were focused on the elaboration of EVOH based nanocomposites in order to increase the material barrier properties of the material. Among the different fillers used in association with EVOH [4,5,6,7,8,9,10,11,12,13,14], literature data reported promising results when incorporating graphene derived fillers in the polymer matrix. Graphene is a highly crystalline material composed of stacks of several one-atom-thick sheets of carbons with a hexagonal structure [15,16,17]. Because of a high shape factor and hydrophobic behavior, graphene derived fillers are widely used to improve the barrier properties of polymer materials [17,18,19,20]. In the majority of the literature data related to EVOH/graphene systems, the elaboration of the nanocomposites was realized through a solution casting process, using dimethyl sulfoxide (DMSO) as a solvent. This process displays the disadvantage of being complicated to scale up for industrial applications, as well as having a high environmental impact [21,22]. To our knowledge, only a few studies on the feasibility of EVOH/graphene-based nanocomposites preparation through a melt blending process have been undertaken [12,23,24]. It appeared that the incorporated fillers were poorly dispersed, mainly because of a low compatibility with the EVOH matrix. Despite the fact that non optimized filler dispersion states were usually obtained, a significant improvement of the barrier properties was observed in some cases [12]. However, it is noteworthy that the vast majority of the studies dedicated to barrier properties focused on measuring the materials performances at low hydration state.

The aim of the present work is to elaborate EVOH based nanocomposites containing 2 wt% graphene derived fillers by a melt blending method. More specifically, four fillers grades going from graphene composed of several sheets to multilayer sheets to graphite under non expanded and expanded forms were used to be compared. A detailed analysis of the filler dispersion state was performed and the impact of fillers on the polymer microstructure, thermal, and mechanical properties was examined. Finally, water vapor and oxygen barrier properties were investigated at high hydration states and related to the nanocomposites morphology, including both filler dispersion state and polymer matrix microstructural changes. The most promising nanocomposite for mechanical and barrier properties was identified. 

## 2. Materials and Methods

### 2.1. Materials

EVOH copolymer (F171B from EVAL Europe, Melsele, Belgium) with 32% mol ethylene and an average molecular weight, $\bar{M_{W}}$ of 30,200 g mol^−1^ was used in this study. Density of the copolymer was equal to 1.19 g.cm^−3^ and its melting temperature ($T_{m_{EVOH}}$) was 182 °C. Four fillers were used in this study. Two fillers were provided from K-Nano, Xiamen, China The first one, KNG-G5, which will be named G5 in this work, is a high-quality graphene composed of aggregates of one to five graphene sheets with a mean diameter (D50) in the range 5–20 μm. The second one, KNG-G150 denoted as G150 in this work, corresponds to stacks of multilayer graphene sheets. The nanoplatelets thickness is lower than 15 nm and the mean diameter (D50) is about 5 µm. The two additional fillers used in this work were of graphite type. They were provided by Imerys, (Paris, France.) TIMREX C-THERM002 is a graphite conditioned in fine powder that will be named CT2 whereas TIMREX C-THERM001 is a graphite powder displaying an expensed shape and it will be denoted as CT1. All the studied fillers underwent no treatment (neither surface modification nor oxidative treatment) before use and presented a density of about 2.2 g.cm^−3^. In order to equilibrate the samples used for mechanical characterization at a defined water activity of *a_w_* = 0.43, the films were placed at least 1 week in a desiccator containing a saturated salt solution of potassium carbonate (K_2_CO_3_ being purchased from Merck, Darmstadt, Germany).

### 2.2. Nanocomposite Films Preparation

Prior to the processing step, the EVOH pellets and filler powder were dried at 90 °C over 48 h to eliminate the residual water. Melt blending was carried out in a Haake internal batch mixer Rheomix 600 (Haake, Vreden, Germany) (mixing chamber of 50 cm^3^) for 10 min at 190 °C using a rotor speed of 50 rpm. The filler content was fixed at 2 wt% for all the nanocomposites. The obtained nanocomposites were then compression molded at 200 °C during 2 min under a pressure of 400 bar to obtain films of about 100 µm. Neat EVOH film used as a reference were prepared according to the same processing conditions.

### 2.3. Morphology Analysis

A Philips CM120 electron microscope (Philips, Amsterdam, The Netherlands) with an accelerating voltage of 120 kV was used for transmission electron microscopy (TEM) analysis. Films were firstly cut at −85 °C with a Leica EM UC7 cryo-ultramicrotome (Wetzlar, Germany) equipped with a diamond knife to obtain ultrathin sections of about 90 nm thicknesses. The samples were placed on Formvar coated grids for observation. Low electron beam intensity and short time of exposure were used for observation to avoid any degradation phenomenon. A quantitative analysis of the size of the dispersed domains was performed thanks to ImageJ software (National Institute of Health, open source).

### 2.4. Thermal Analysis

Differential scanning calorimetry (DSC) analyses were performed with a TA Instruments DSC QA 200 (Waters Corporation, Milford, MA, USA), on 5–10 mg samples placed in hermetic pans. Two consecutive heating scans were carried out from −50 °C to 250 °C at 10 °C·min^−1^ with an intermediate cooling step at 10 °C·min^−1^. The melting temperature (*T_m_*), crystallization temperature (*T_c_*) and glass transition temperature (*T_g_*) were measured during the second scan. The crystalline index was calculated according to the following equation:

(1) $$X_{c}\;\left(\%\right)=\frac{\Delta H_{EVOH}}{1-w_{filler}}/\;\Delta H_{EVOH}^{\infty }$$

where $\Delta H_{EVOH}$ and $\Delta H_{\mathrm{EVOH}}^{\infty }$ are the measured and theoretical 100% crystalline melting enthalpy of EVOH respectively, and *w_filler_* is the weight fraction of the fillers incorporated in the EVOH matrix. The theoretical 100% crystalline melting enthalpy of EVOH32 $\left(\Delta H_{EVOH}^{\infty }\right)$, calculated from the additive law proposed by Rwei et al. was equal to 188.2 (J·g^−1^) [25].

Thermogravimetric analysis (TGA) was carried out with a TA Instruments TGA Q500 (Waters Corporation, Milford, MA, USA). Results were collected under dry helium atmosphere on samples of 25–35 mg during a ramp of temperature from 25 to 600 °C performed at 20 °C·min^−1^. The weight loss curves and first derivate weight loss curves were analyzed and the degradation temperatures (*T_5%_*) and (*T_max_*), corresponding to a mass loss of 5 wt% and the maximum of the peak of the first derivate weight loss curve respectively, were determined.

### 2.5. Tensile Tests

Uniaxial tensile tests were performed at 23 °C on H3 type tensile specimens (total length: 50 mm—useful length: 10 mm—useful width: 4 mm) equilibrated during at least 1 week at *aw* = 0.43. It was checked that this duration allowed each sample to reach water sorption equilibrium. The mechanical tests were carried out with a CRITERION (MTS Systems, Créteil, France) testing machine equipped with a 500 N load cell at a cross-head speed of 10 mm·min^−1^. The gauge length was fixed at 10 mm. Values of tensile modulus (*E*), yield stress (*σ_e_*), yield deformation (*ε_e_*), breaking stress (*σ_r_*), and deformation at break (*ε_r_*) were determined from the stress–elongation curves. The reported values of the mechanical characteristics were the arithmetic mean of at least three different specimens. The precision on the values of the mechanical parameters was estimated to be better than 10%. 

### 2.6. Water Permeability

Water permeability measurements were performed with a Mocon Permatran W 3/33 (Minneapolis, MN, USA) equipped with an infrared sensor. The permeation cell was constituted of two chambers separated by the film to be tested. The film testing area was 50 cm^2^. Prior to testing, specimens were conditioned in nitrogen inside the cell for at least 12 h. Then, water molecules were introduced in the upstream compartment of the permeation cell. The water molecules transferred through the film were conducted by the carrier gas (N_2_) to the infrared sensor. The water permeability coefficient ($P_{H_{2}O}$) was calculated considering the following equation:

(2) $$P_{H_{2}O}=\frac{J_{st}{}_{H_{2}O}\cdot L}{\Delta p}$$

where *L* is the thickness of the film, $J_{st}{}_{H_{2}O}$ the water stationary flux and Δ*p* the difference of pressure between the upstream, and the downstream compartments. $P_{H_{2}O}$ was expressed in Barrer (1 Barrer = 10^−10^ cm_STP_^3^·cm·cm^−2^·s^−1^·cm_Hg_^−1^ = 3.36 × 10^−16^ mol·m·m^−2^·s^−1^·Pa^−1^). Measurements were performed at controlled temperature (*T* = 25 °C) for a water activity of 1. The precision on the values of the permeability coefficient was estimated to be better than 5%.

### 2.7. Oxygen Permeability

Oxygen permeability measurements were performed on a Mocon Oxtran 2/21 (Mineapolis, MN, USA) equipped with a Coulox sensor. The permeation cell was composed of two compartments separated by the film to be studied. The sample area was 50 cm^2^. Nitrogen containing 2% of hydrogen (N_2_/H_2_) was used as the carrier gas and pure oxygen was used as the test gas. The water activity of the two gases was controlled by a humidifier. Each sample was conditioned within the permeation cell for at least 24 h prior to testing, with the objective to remove traces of atmospheric oxygen and to reach the film water sorption equilibrium. Then, oxygen was introduced in the upstream compartment of the test cell. Oxygen transferred through the film was conducted by the carrier N_2_/H_2_ gas to the coulometric sensor. The oxygen permeability coefficient $\left(P_{O_{2}}\right)$ was calculated considering the following equation:

(3) $$P_{O_{2}}=\frac{J_{st}{}_{O_{2}}\cdot L}{\Delta p}$$

where *L* is the thickness of the film, $J_{st}{}_{O_{2}}$ the oxygen stationary flux and Δ*p* the difference of pressure between the upstream and the downstream compartments. $P_{O_{2}}$ values were expressed in Barrer. Measurements were performed at controlled temperature (*T* = 25 °C) and for a water activity of 0.95. The precision on the values of the permeability coefficient was estimated to be better than 5%.

## 3. Results

The influence of the filler type on the morphology, thermal, mechanical, and barrier properties of the nanocomposites was investigated with the aim to establish interrelationships between the structure and the functional properties.

### 3.1. Morphology

**Figure 1:**
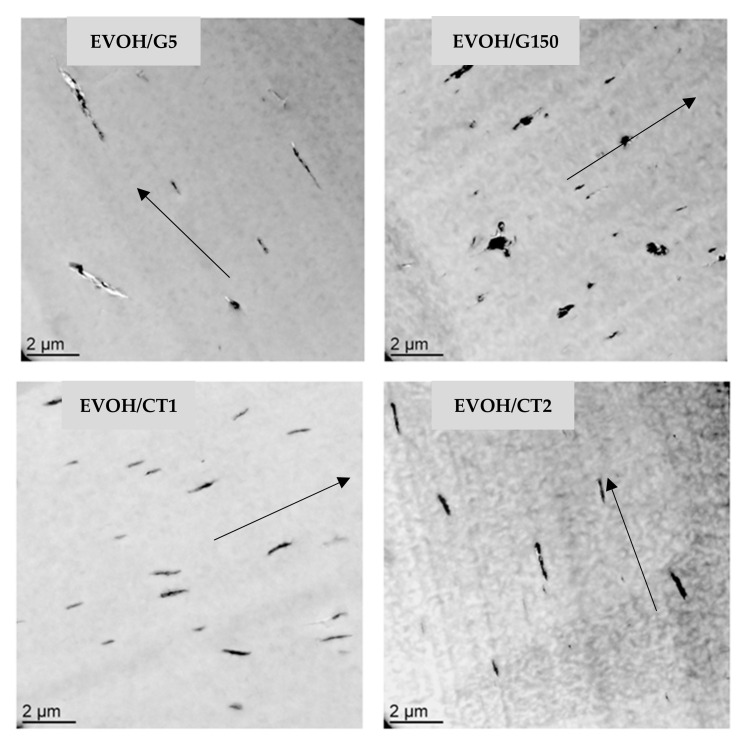
TEM micrographs at low magnifications of the studied nanocomposites as a function of the filler type (filler rate = 2 wt%)—the arrows indicate the preferential orientation of the fillers which is parallel to the film surface.

**Figure 2:**
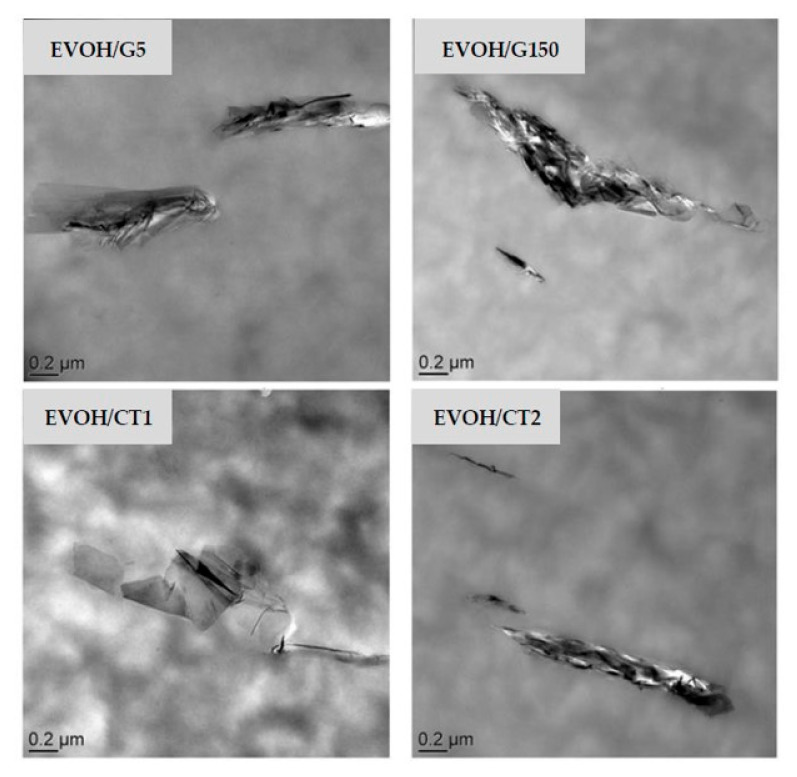
TEM micrographs at high magnifications of the studied nanocomposites as a function of the filler type (filler rate = 2 wt%).

To analyze the nanocomposites morphology, TEM analysis was performed at different magnifications and characteristic TEM images of the samples are shown in Figure 1 and Figure 2. Figure 1 revealed that the fillers were lying in the plane of the film for all samples. Although all samples exhibited a homogeneous dispersion of small filler stacks, EVOH/G150 seemed to display filler domains with the lowest aspect ratio.

In order to evaluate more precisely the fineness of the filler dispersion within the EVOH matrix, analyses were also performed through high magnification. The images shown in Figure 2 allowed confirming the presence of dispersed platelet stacks in all samples. It was also confirmed that stacks were thicker for EVOH/G150. It is noteworthy that single-layer and few layer graphene sheets were clearly observed in EVOH/CT1 sample in addition to small stacks. 

**Figure 3:**
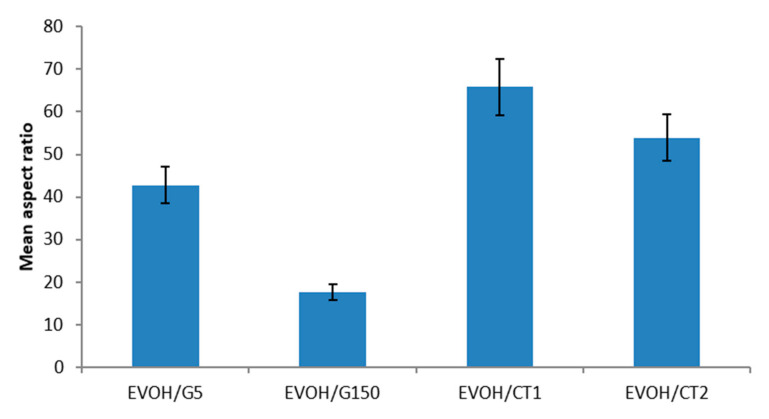
Comparison of the mean average aspect ratio of the filler as a function of the studied nanocomposite.

A quantitative analysis of the size of the dispersed domains was performed for each nanocomposite to determine the filler mean aspect ratio (α). The obtained results are reported in Figure 3.

Significant differences were observed between the studied systems. Indeed, α value was the lowest for EVOH/G150, with a value of about 20. EVOH/G5 nanocomposite exhibited an aspect ratio value of about 45, while the highest values were obtained for the EVOH/CT1 and EVOH/CT2 films, with a mean aspect ratio of 70 and 55 respectively. 

These results demonstrated the ability of graphene sheets based fillers to be dispersed in an EVOH matrix by a melt blending, contrary to most of the literature studies related with the elaboration of similar nanocomposites by solvent casting process that evidenced the presence of high size filler stacks [12,26,27]. Moreover, our results underlined the interest of the graphite filler type under an expanded shape as it led to the finest platelet dispersion and the highest mean aspect ratio.

### 3.2. Thermal Properties

**Figure 4:**
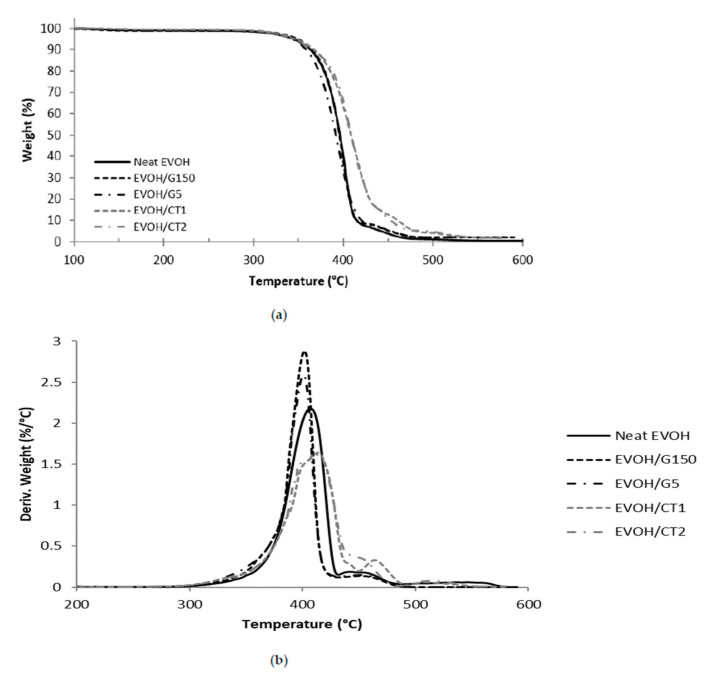
Representation of (**a**) weight loss curves and (**b**) first derivative weight loss curves of neat EVOH and studied nanocomposites (20 °C·min^−1^, filler rate = 2 wt%).

The thermal properties of the nanocomposites were investigated by TGA analysis. The weight loss curves of neat EVOH and nanocomposites samples and their derivatives are presented in Figure 4.

The neat EVOH sample exhibited two steps of degradation. Indeed, the degradation of the hydroxyl groups of the polymer occurred between 310 °C and 420 °C while the degradation situated around 450 °C was related to the decomposition of ethylene segments into carbon chains [28]. The residue at high temperature was equal to 0.

EVOH/G150 and EVOH/G5 nanocomposites displayed similar behavior than the neat EVOH, except for the residue which was equal to 2 wt% and corresponded to the filler content. For EVOH/CT1 and EVOH/CT2, the mass loss curve was shifted to higher temperature and the measured residue was also equal to 2 wt% (Figure 4a).

In order to get a better understanding of the thermal stability of the films, the degradation temperatures *T_5%_* corresponding to a decrease of the sample mass of 5 wt% and *T_max_* taken at the maximum of the main peak observed in the derivative curve were determined. The values obtained from Figure 4b were reported in Table 1.

For the neat EVOH, *T_5%_* and *T_max_* values were equal to 344 °C and 407 °C respectively, which is in agreement with the literature [24,29,30]. For the nanocomposites, similar values of *T_5%_* were obtained, with temperatures between 344 °C and 349 °C. The same tendency was noticed by George and Bhowmick with the study of EVA based nanocomposites containing 2 wt% of expanded graphite [31]. This showed that the polymer degradation began at the same temperature whatever the studied material.

The analysis of *T_max_* values showed that incorporation of G150 and G5 slightly decreased *T_max_* with respect to neat EVOH (Δ*T_max_* = −5 °C). On the other hand, adding CT2 or CT1 fillers within the EVOH matrix led to increase *T_max_* from 407 °C to 411 and 414 °C, respectively. These results suggested a slight increase of the degradation rate for EVOH/G150 and EVOH/G5 and a slowing down of the degradation rate for EVOH/CT2 and EVOH/CT1 nanocomposites. The enhanced thermal stability observed when using CT1 and CT2 graphites could be explained by the inherent high thermal stability of the fillers and their good dispersion within the EVOH matrix which delayed the polymer chains degradation. Such trend was already observed in literature; however, the nanocomposites were prepared in these cases by a solvent blending process [9,32].

### 3.3. Microstructure

**Figure 5:**
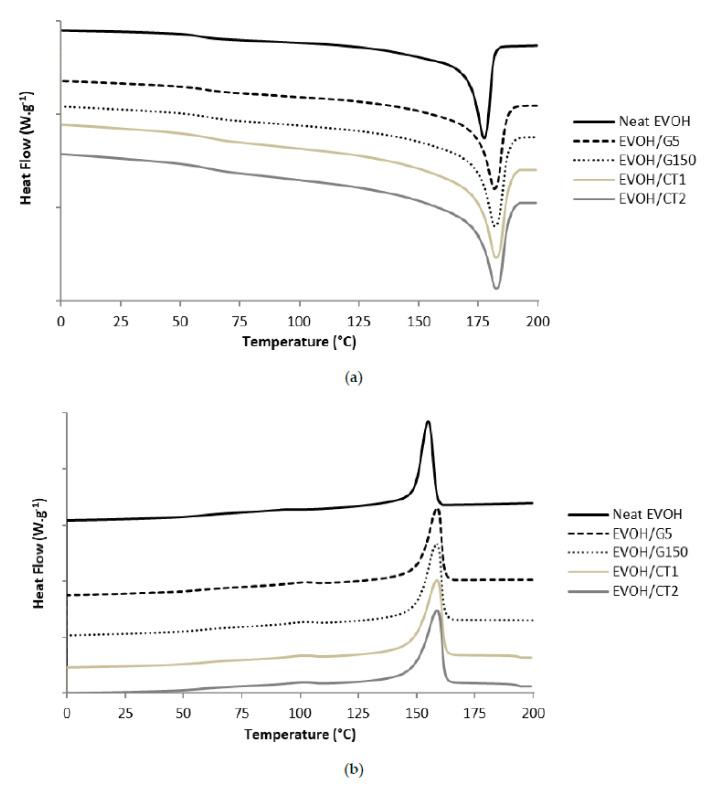
DSC (**a**) heating and (**b**) cooling thermograms of neat EVOH and studied nanocomposites (scanning rate = 10 °C·min^−1^, *a_w_* = 0, shifted curves).

DSC analyses were performed in order to determine the influence of the filler incorporation on the polymer matrix microstructure. The obtained thermograms are presented in Figure 5 and *T_g_*, *T_m_*, *T_c_*, and *X_c_* values are detailed in Table 2. 

It could be noted that the *T_g_*, *T_m_*, and *T_c_* values measured on the four different nanocomposites were slightly higher than those obtained on the neat matrix [27,33]. The increase observed for *T_c_* showed that the nanofillers could behave as nucleating agents for the EVOH matrix while the shift of *T_m_* suggested that the polymer crystalline lamellae formed were thicker in the presence of the nanofillers.

Moreover, it appeared that the crystallinity of the nanocomposites was significantly higher than the one of the neat EVOH, with an increase more pronounced (by a factor close to 1.3) for the EVOH/CT1 and EVOH/CT2 samples (*X_c_* = 49% and 50% respectively compared to a value of 39% for the neat EVOH). The same tendency was observed by Kwon et al. with the increase of a factor up to 2 of the crystallinity with the incorporation of 1 wt% of highly exfoliated graphene to an EVOH32 matrix by a solution blending method [12]. These results were explained by the presence of high surface areas of graphene sheets which could behave like nucleating agents for the EVOH matrix [34]. For our nanocomposites series, it could be assumed that the surface areas of fillers in contact with the EVOH matrix was higher for EVOH/CT2 and EVOH/CT1 due to an enhanced filler dispersion state.

### 3.4. Mechanical Properties

**Figure 6:**
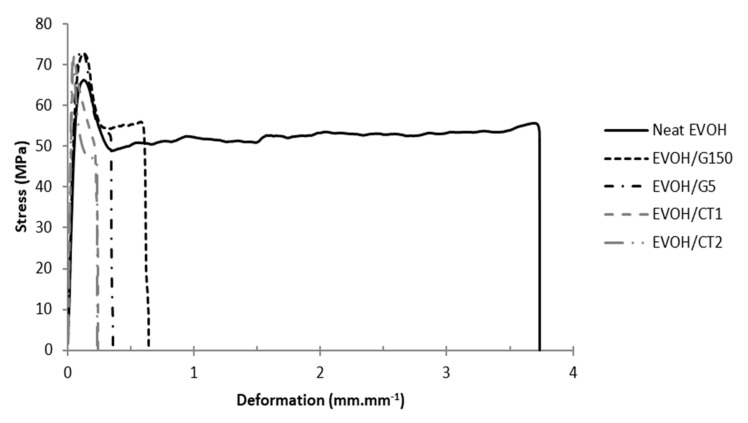
Representative stress-deformation curves of neat EVOH and EVOH nanocomposites samples (*a_w_* = 0,43, *T* = 25 °C, filler rate = 2 wt%).

Representative stress-strain curves obtained at a water activity of 0.43 are presented in Figure 6 for each studied material. The Young modulus (*E*), yield stress (*σ_e_*), yield deformation (*ε_e_*), breaking stress (*σ_r_*) and deformation at break (*ε_r_*) values are reported in Table 3. 

The neat EVOH film displayed a Young modulus of 1070 MPa; the yield stress and breaking stress were noticed at 69 MPa and 49 MPa respectively, while the yield deformation and the deformation at break occurred at values close to 0.14 and 3.77 mm·mm^−1^ respectively, in agreement with literature data [35,36].

A significant increase of the Young modulus was observed for the nanocomposites with values around 1200 MPa for samples containing G150 and G5 filler, 1370 MPa for the EVOH/CT2 film, and up to 1430 MPa for the EVOH/CT1 film. This trend, which is in agreement with the one generally observed for nanocomposites [33,37], could be explained by the high aspect ratio of graphene sheet, the alignment of the fillers in the plane of the film and a significant stress transfer from the matrix to the incorporated fillers [38,39,40]. Moreover, the highest increase of Young modulus was obtained for the EVOH/CT1 system thanks to an enhanced filler dispersion state. 

The yield stress *σ_e_* remained similar whatever the material composition with values close to 70 MPa while the tensile strength *σ_r_* slightly increased for all nanocomposites compared to the neat EVOH. The same tendency was noted by Barreira et al. with the incorporation of 0.5 wt% graphene oxide in an EVOH32 matrix by a melting process [24].

If EVOH/G5 and EVOH/G150 nanocomposites exhibited similar yield deformation values than neat EVOH, a decrease by a factor close to 2 of the yield deformation, was observed going from neat EVOH to EVOH/CT1 and EVOH/CT2. 

Finally, it appeared that the deformation at break significantly decreased for the nanocomposites compared to the neat EVOH, in accordance with literature data [5,11,41,42,43]. The deformation decrease was lower for EVOH/G150 filler in comparison with EVOH/G5, EVOH/CT1, and EVOH/CT2 nanocomposites.

The mechanical properties of the nanocomposites could be related to the strengthening effects brought by the fillers and the matrix crystalline phase. The highest Young modulus and lowest deformation at break were obtained for EVOH/CT1 which exhibited an important increase of the EVOH crystallinity degree and the highest filler aspect ratio due to the coexistence of dispersed single-layer and few layer graphene sheets.

### 3.5. Water and Oxygen Barrier Properties

**Figure 7:**
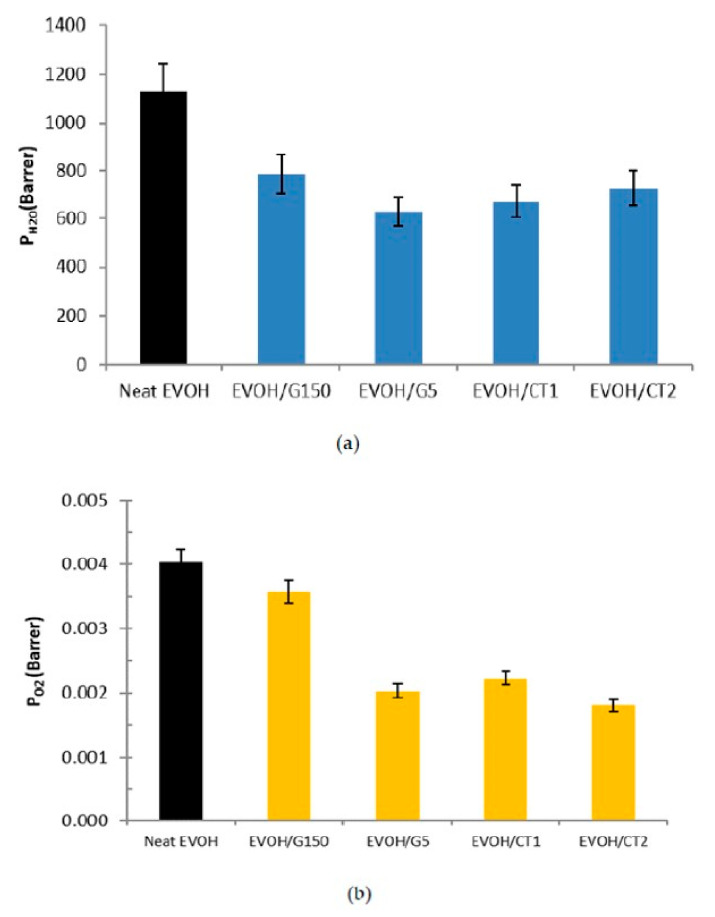
Comparison of the (**a**) water and (**b**) oxygen permeability coefficients of the neat EVOH and studied nanocomposites (*T* = 25 °C, *a_w_* = 1, filler rate = 2 wt%).

Because of the high moisture sensitivity of the EVOH copolymers, a significant loss of their barrier properties is observed at high water activities [2,35,36,44,45]. However, most of the studies from literature were only focused on the improvement of the barrier properties at low water activities through the elaboration of nanocomposites [41,46,47,48,49]. It has to be pointed out that different studies showed that the barrier gain obtained for nanocomposites at low relative humidity was not maintained at high water activities [50,51]. Moreover, no work was completed which studied both water and O_2_ permeability. As a consequence, in this work the water barrier properties of the films were determined at a critical water activity *a_w_* = 1 and oxygen permeability was investigated at a relative humidity of 0.95. The results are presented in Figure 7a,b.

Concerning water permeability, the neat EVOH sample exhibited a water permeability value of about 1150 Barrer, which is in accordance with the results obtained in literature [52].

For the nanocomposites, a significant improvement of the water barrier properties was observed. Indeed, the water permeability coefficient decreased down to a value around 650 Barrer for EVOH/CT1 and EVOH/G5.

The observed permeability decrease (by a factor 1.8) was significantly higher than the one reported by Kwon et al., who underlined a decrease by a factor 1.4 at *a_w_* = 0.9 of the water vapor permeabilities for EVOH32 nanocomposites elaborated by solution blending and containing 2 wt% of exfoliated graphite nanosheets [26]. It is noteworthy that the permeability decrease was only slightly lower for EVOH/CT2 ($P_{H_{2}O}$= 730 Barrer) in comparison with EVOH/CT1 and EVOH/G5 ($P_{H_{2}O}$= 650 Barrer) and much lower for EVOH/G150 ($P_{H_{2}O}$= 790 Barrer) which exhibited the lowest filler mean aspect ratio.

The same trends were observed for oxygen permeability coefficients, G150 filler leading to the lowest increase of gas barrier properties whereas G5, CT1, and CT2 fillers allowed to decrease the oxygen permeability coefficient by a factor close to 2. These results were in agreement with those obtained by Kwon et al. [26], who have shown a decrease of the oxygen permeabilities by a factor close to 3 but for a water activity of 0.8 (lower than the one studied in this work) with the elaboration by a melting process of nanocomposites based on EVOH and 2 wt% of exfoliated graphite.

Thus, these results suggested that the incorporation of graphene-based fillers in the EVOH copolymer allowed to improve the water and oxygen barrier properties of the studied films even at a high water activity (*a_w_* > 0.9).

In order to analyze the influence of the fillers on the barrier properties, the relative permeability (e.g., the permeability of the nanocomposites ratioed to the permeability of the neat matrix) was calculated for each nanocomposite. The relative permeability values were compared to the theoretical values obtained from the most widely used analytical model, that considers nanocomposites with fillers aligned in the plane of the film, which corresponds to the morphology evidenced for all the studied nanocomposites [53].

(4) $$\frac{P}{P_{0}}=\frac{1-\phi_{v}}{1+\alpha \cdot \frac{\phi_{v}}{2}}$$

where *P*/*P*_0_ is the permeability ratio of the nanocomposite and the neat polymer, *ϕ_v_* the volume fraction of the incorporated filler and *α* the filler aspect ratio.

**Figure 8:**
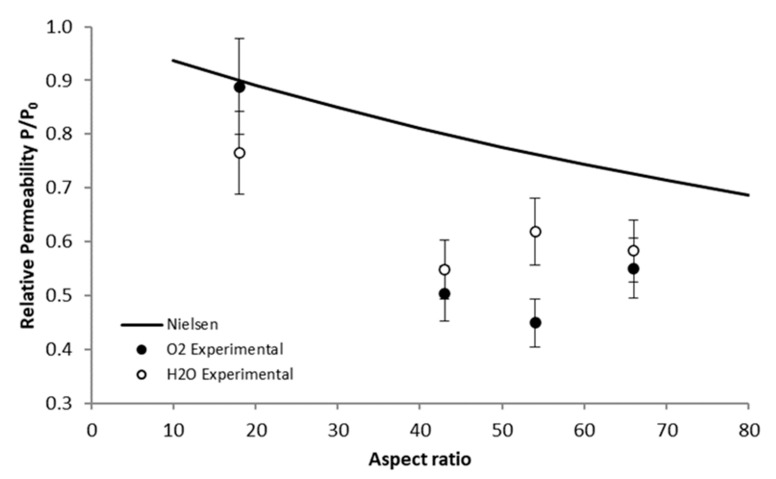
Comparison of the relative permeability of the studied nanocomposites with the Nielsen predictive law (*T* = 25 °C, *a_w_* = 1, filler rate = 2 wt%).

The experimental values of the relative permeability calculated for oxygen and water permeability, respectively were compared to the theoretical values represented by the full line in Figure 8.

Firstly, taking into account the uncertainty on the permeability values, it could be observed that there is good agreement between the experimental relative permeability values of the two permeant molecules. It could also be underlined that the values obtained from the Nielsen model were notably higher than the experimental ones, less pronounced for the EVOH/G150 system. The overestimation of the values by the model could reveal that the decrease of the permeability was not only attributed to the effect of dispersed fillers.

Indeed, as seen from the microstructure analysis, it appeared that the incorporation of graphene led to a significant increase of the EVOH crystallinity. In other words, the presence of the fillers allowed to reduce the amorphous part of the matrix and thus to increase the amount of impermeable regions. The results also demonstrated that the crystallinity of the elaborated nanocomposites varied with the type of incorporated fillers. A study from Picard et al. underlined that an additional effect of the crystallinity could be observed on the permeabilities of nanocomposites [54].

In order to evaluate the sole impact of the modification of polymer crystallinity on the barrier properties, Hardy et al. [55] proposed to use the following equation derived from the Maxwell law [56]:

(5) $$\frac{P_{0}\prime }{P_{0}}=\left(\frac{1-X_{c0^{\prime }}}{1-X_{c0}}\right)\left(\frac{1+\left(\frac{X_{c0}}{2}\right)}{1+\left(\frac{X_{c0^{\prime }}}{2}\right)}\right)$$

with *P*_0_ and *P*_0_*′* the permeabilities of the neat EVOH and the EVOH matrix in the nanocomposites respectively, $X_{c_{0}}$ and ${X_{c_{0}}}^{\prime }$ the corresponding crystallinity values.

Taking account of the additional effect of the presence of the filler in the EVOH matrix using the Nielsen law, it is possible to obtain the equation:

(6) $$\frac{P}{P_{0}}=\left(\frac{1-\phi_{v}}{1+a\frac{\phi_{v}}{2}}\right)P_{0}\prime /P_{0}$$

where *P* is the nanocomposite permeability, α the mean filler aspect ratio, and *ϕ_v_* the volume fraction of fillers.

By replacing *P*_0_*′*/*P*_0_ ratio by its developed equation (Equation (5)), it was possible to express the relative permeability of the elaborated nanocomposites taking into account the simultaneous contribution of the presence of the incorporated fillers and the increase of the matrix crystallinity:

(7) $$\frac{P}{P_{0}}=\left(\frac{1-X_{c0^{\prime }}}{1-X_{c0}}\right)\left(\frac{1+\left(\frac{X_{c0}}{2}\right)}{1+\left(\frac{X_{c0^{\prime }}}{2}\right)}\right)\left(\frac{1-\phi_{v}}{1+a\frac{\phi_{v}}{2}}\right)$$

The detailed values obtained from the different models were reported in Table 4.

The data obtained from the Maxwell model appeared higher than the experimental values, which also underlined that the increase of the crystallinity of the EVOH matrix was not sufficient to explain the decrease of the permeabilities, similarly to the sole effect of the presence of fillers with a high aspect ratio.

However, the predicted permeability ratios obtained from Equation (7) revealed a good accordance with the experimental values.

These results suggested that the improvement of the barrier properties could be assigned to the simultaneous contribution of the presence of high aspect ratio fillers and of the increase of the EVOH matrix crystalline degree.

## 4. Conclusions

Nanocomposites based on an EVOH matrix and different graphene type fillers were successfully elaborated by a melt blending method for a filler content equal to 2 wt%. Indeed, the morphology analyses demonstrated the feasibility to correctly disperse graphene sheets based fillers in an EVOH matrix by this process. The micrographs revealed the presence of well dispersed particles with a favored orientation parallel to the surface of the film. The coexistence of single-layer and few layers graphene sheets was observed for the nanocomposite prepared with the graphite filler displaying an expanded powder shape.

An improvement of the thermal stability was evidenced for the nanocomposites prepared with the graphite fillers. A significant increase of the matrix crystallinity was noticed upon filler incorporation, whatever the filler type. The crystallinity increase was more pronounced when using the graphite type fillers. An increase of the Young modulus and a decrease of the deformation at break was evidenced for all nanocomposites. This behavior could be attributed to the high aspect ratio of the fillers and the restricted movement of the polymer chains in the presence of these platelets.

Finally, analyses of the water vapor and oxygen barrier properties at high relative humidity evidenced a significant decrease of the permeabilities for the nanocomposites, more pronounced for the samples displaying the best quality of filler dispersion and the highest crystallinity index. Thus, the elaborated nanocomposites systems presented a high improvement of the barrier properties with a decrease by a factor close to 1.7 of the water permeabilities and a decrease by a factor close to 2 of the oxygen permeabilities. As evidenced with a model derived from Nielsen and Maxwell laws, this tendency could be explained by the increase of the diffusion path of the permeant molecules through a tortuosity effect induced by the presence of oriented graphene sheets presenting a high aspect ratio and the increase of the amount of impermeable phase within the matrix due to the increase of its crystallinity degree. By combining the finest platelet dispersion state and a significant increase of the EVOH matrix crystallinity, EVOH/CT1 exhibited the highest Young modulus and an interesting increase of thermal stability, water, and oxygen barrier properties. 

## Figures and Tables

**Table 1:** Comparison of *T_5%_* and *T_max_* values as a function of the studied EVOH/Graphene nanocomposite (the uncertainty is better than 1 °C).

Sample	Neat EVOH	EVOH/G150	EVOH/G5	EVOH/CT1	EVOH/CT2
***T_5%_*** **(°C)**	344	344	349	346	346
***T_max_*** **(°C)**	407	402	402	414	411

**Table 2:** Comparison of the glass transition temperature (*T_g_*), *melting temperature (T_m_*), crystallization temperature (*T_c_*), and crystallinity degree (*Xc*) as a function of the studied nanocomposite (*a_w_* = 0, filler rate = 2 wt%) (the uncertainty is better than 1 °C) (*a_w_* = 0, filler rate = 2 wt%).

Sample	Neat EVOH	EVOH/G150	EVOH/G5	EVOH/CT1	EVOH/CT2
*T_g_* (°C)	60	62	61	62	62
*T_m_* (°C)	178	182	182	183	183
*T_c_* (°C)	156	159	159	159	159
*X_c_* (%)	39	43	45	49	50

**Table 3:** Comparison of the mechanical properties of the studied nanocomposites. (*a_w_* = 0.43, *T* = 25 °C, filler rate = 2 wt%)

Sample	E (MPa)	*σ_e_* (MPa)	*ε_e_* (mm·mm^−1^)	*σ_r_* (MPa)	*ε_r_* (mm·mm^−1^)
**Neat EVOH**	1070 ± 110	69 ± 7	0.14 ± 0.01	49 ± 5	3.77 ± 0.4
**EVOH/G150**	1200 ± 120	72 ± 7	0.12 ± 0.01	56 ± 6	0.82 ± 0.08
**EVOH/G5**	1200 ± 120	68 ± 7	0.11 ± 0.01	53 ± 5	0.25 ± 0.03
**EVOH/CT1**	1430 ± 140	72 ± 7	0.08 ± 0.01	58 ± 6	0.16 ± 0.02
**EVOH/CT2**	1370 ± 140	69 ± 7	0.07 ± 0.01	55 ± 6	0.17 ± 0.02

**Table 4:** Modeling of the relative permeability of the nanocomposites containing 2 wt% of filler (*a_w_* = 0.95, *T* = 25 °C).

*P*/*P*_0_	EVOH/G150	EVOH/G5	EVOH/CT1	EVOH/CT2
	**Experimental**			
H_2_O	0.77 ± 0.08	0.55 ± 0.06	0.58 ± 0.06	0.62 ± 0.06
O_2_	0.89 ± 0.09	0.50 ± 0.05	0.55 ± 0.06	0.45 ± 0.05
	**Models**			
Nielsen (Equation (4))	0.90 ± 0.09	0.80 ± 0.08	0.73 ± 0.07	0.76 ± 0.08
Maxwell (Equation (5))	0.92 ± 0.09	0.90 ± 0.09	0.80 ± 0.08	0.78 ± 0.08
Nielsen + Maxwell (Equation (7))	0.83 ± 0.08	0.69 ± 0.07	0.58 ± 0.06	0.60 ± 0.06

## Data Availability

Data are available on request.
